# HyperQuaternionE: A hyperbolic embedding model for qualitative spatial and temporal reasoning

**DOI:** 10.1007/s10707-022-00469-y

**Published:** 2022-09-05

**Authors:** Ling Cai, Krzysztof Janowicz, Rui Zhu, Gengchen Mai, Bo Yan, Zhangyu Wang

**Affiliations:** 1 Center for Spatial Studies, CA UC Santa Barbara, USA; 2 Computer Science Department, Stanford University, CA Stanford, USA; 3 Department of Geography and Regional Research, University of Vienna, Vienna, Austria; 4 School of Geographical Sciences, University of Bristol, Bristol, United Kingdom

**Keywords:** Qualitative spatial and temporal reasoning, Hyperbolic embeddings, Composition table, Conceptual neighborhood

## Abstract

Qualitative spatial/temporal reasoning (QSR/QTR) plays a key role in research on human cognition, e.g., as it relates to navigation, as well as in work on robotics and artificial intelligence. Although previous work has mainly focused on various spatial and temporal calculi, more recently representation learning techniques such as embedding have been applied to reasoning and inference tasks such as query answering and knowledge base completion. These subsymbolic and learnable representations are well suited for handling noise and efficiency problems that plagued prior work. However, applying embedding techniques to spatial and temporal reasoning has received little attention to date. In this paper, we explore two research questions: (1) How do embedding-based methods perform empirically compared to traditional reasoning methods on QSR/QTR problems? (2) If the embedding-based methods are better, what causes this superiority? In order to answer these questions, we first propose a hyperbolic embedding model, called HyperQuaternionE, to capture varying properties of relations (such as symmetry and anti-symmetry), to learn inversion relations and relation compositions (i.e., composition tables), and to model hierarchical structures over entities induced by transitive relations. We conduct various experiments on two synthetic datasets to demonstrate the advantages of our proposed embedding-based method against existing embedding models as well as traditional reasoners with respect to entity inference and relation inference. Additionally, our qualitative analysis reveals that our method is able to learn conceptual neighborhoods implicitly. We conclude that the success of our method is attributed to its ability to model composition tables and learn conceptual neighbors, which are among the core building blocks of QSR/QTR.

## Introduction

In our daily life, we humans usually use qualitative expressions, such as *left*, *north*, *after* and *during*, to describe and infer spatial/temporal relations between two objects. The field that studies how to enable machines/artificial intelligence (AI) agents to represent qualitative spatial and temporal expressions, and to draw inferences on top of these representations, namely qualitative spatial/temporal reasoning (QSR/QTR), is an active research topic in AI. In the past years, it has fostered a variety of research across various applications such as cognitive robotics [1], visual sensemaking [2], semantic question answering  [3], spatio-temporal data mining [4] and (spatial) cognition and navigation [5, 6].

**Fig. 1:**
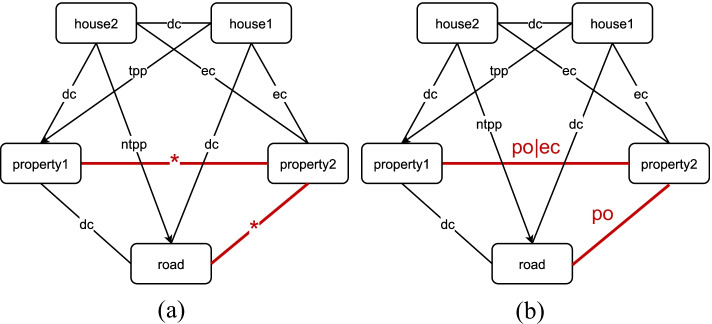
Constraint network-based reasoning. The symbol $*$ in red denotes all RCC-8 relations. Full names of relations are described in Table 1. Figure 1(**a**) illustrates the initial constraints between entities imposed by the relations on edges, and Fig. 1(**b**) shows the resulting relations after path-consistency checking

Since the late 1980s, a plethora of theoretical research have been dedicated to computational QSR/QTR [7–16]. Among them two best studied fundamental problems in qualitative reasoning (QR) are qualitative knowledge *representation* and *reasoning*. In the past, a lot of work has focused on the knowledge representation aspect. For instance, non-null regions in an n-dimensional embedding space $\mathbb {R}^n$ [17] are taken as ontological primitives, and binary topological relations, i.e., Region Connection Calculus (RCC)-8 relations [8, 18], and Allen’s temporal relations [7] as primitive relations between two regions/time intervals. Reasoning, however, remains to be a challenge. Composition tables (CT) and conceptual neighborhood structures (CNS) are among the major reasoning techniques, jointly supporting inferences about spatial and temporal relations between geospatial entities or events [5, 8, 19, 20]. For instance, one can use CT as constraints to reason over spatial relations. Simply put, such a method regards known binary relations as constraints between regions. Then the reasoning task boils down to a consistency satisfactory problem (CSP), i.e., to determine whether the available information is consistent or not, given the CT. For example, as shown in Fig. 1, the possible topological relation between *property1* and *property2* is either *partially overlap* or *externally connected* after path-consistency checking built up on RCC-8’s composition table [21, 22].

Despite those success stories of traditional QR approaches, several limitations remain. First, constraint-based methods are prone to erroneous information, e.g, introduced by noise. Errors may occur at any stage during information collection, and, thus, are inevitable in reality, which may break down the traditional reasoning capabilities. For instance, if the relation between *house2* and *property2* is wrongly recorded as *dc* instead of *ec*, inferring unknown relations based on CT will fail. Second, traditional QR approaches are only applicable to a limited number of reasoning tasks, such as deducing new knowledge, checking consistency, updating existing knowledge, and discovering a minimal set of useful representations. Albeit seemingly different, all these tasks are in fact mutably transformable and can be solved essentially in a similar fashion [23]. Such a shortage of applications is partially attributed to the symbolic knowledge representation used in traditional QR, which prohibits it from being beneficial to other tasks which purely rely on numeric computations. Meanwhile, the symbolic representation of knowledge is usually in the form of triples (i.e., $\langle subject, relation, object \rangle$). Traditional QR approaches only make full use of pairwise constraints between entities while failing to benefit from higher-order interactions. Third, reasoning over spatial/temporal calculi is NP-complete [21], which makes traditional QR methods difficult to scale. Extra efforts (e.g., identifying maximal tractable subsets containing all basic relations and different optimizing strategies) are needed to improve the efficiency, which becomes even more problematic with an increasing number of relations. These limitations, consequently, necessitate more robust spatial/temporal reasoners.

The past decade has witnessed great breakthroughs in Machine Learning (ML). Embedding/sub-symbolic techniques, in particular, have been applied to tackle various reasoning tasks. Examples include word/sentence similarity measuring [24–26], question/query answering [27–30], dynamic interaction inference [31], as well as knowledge graph completion and reasoning [32–35]. Generally speaking, their success can be attributed to learnable sub-symbolic representations (i.e., embeddings) in contrast to symbolic representations. At training, an embedding method is trained to draw patterns of and interactions between entities from data and sub-symbolic representations of entities are learned accordingly. This training process is analogous to knowledge abstraction, which preserves the core essentials of entities but ignores subtle details. Moreover, such a process of automatic abstraction makes embedding models less prone to local errors and data incompleteness, and improve their generability [36, 37].

Despite their appealing characteristics, the adoption of sub-symbolic approaches to QSR/QTR remains mostly unexplored. To fill in this gap, we propose a hyperbolic embedding model, called HyperQuaternionE, as an implicit reasoner for spatial and temporal reasoning. In the model design, we consider the following two prominent characteristics of spatial/temporal reasoning. First, composition tables, which specify role chains of relations, have been the backbone of most qualitative reasoning methods. In order to enable embedding models to automatically find and take use of such role chains, we introduce quaternions, an extension of complex numbers, in the embedding space. Quaternion mutiplication follows the non-commutative law and thus is well suited for modeling relation composition. Additionally, quaternions can be used to model other properties of relations (e.g., symmetric and anti-symmetric) and inverse relations. Second, hierarchical structures over entities must be considered. Certain spatial and temporal relations, such as *non-tangentially proper par* and *before*, are transitive, thus inducing hierarchical structures over entities (e.g., regions or temporal intervals). This suggests that a hyperbolic embedding space, which can embed trees with arbitrarily low distortion [38], would be more appropriate than Euclidean space. Therefore, we adopt hyperbolic space as our embedding space and transfer quaternions to this space to preserve the properties mentioned above. We evaluate our method on two tasks, namely entity inference and relation inference, which are to identify entities that have a given (spatial/temporal) relation (e.g., *partially overlapping*) to a target entity, and to infer the relation held between two given entities, respectively. Finally, we conduct a qualitative analysis over the trained models in order to uncover the reasoning mechanisms behind our model.

The remainder of this paper is structured as follows. Section 2 introduces important concepts and terms applied in the proposed method. Section 3 summarizes related work on spatial and temporal reasoning, knowledge graph embedding models, and their applications in geospatial knowledge graphs. Section 4 elaborates on the motivation of our proposed embedding model and its formulation. To compare the reasoning ability of different models, Section 5 presents the datasets, baseline methods, as well as evaluation metrics used in the study, followed by an experimental summary of key findings. Section 6 concludes our work and points out future research directions.

## Background

Before reviewing related work, we first introduce concepts and terms used in the literature.

### Basic definitions

#### Definition 1

(Spatial and Temporal Relations) In this paper, we focus on the eight topological relations of RCC-8 [8], and the thirteen temporal relations developed by Allen [7]. Table 1 and 2 list those relations together with their inherent properties (i.e., transitive and symmetric).

**Table 1:** List of spatial relations

Name (abbrev.)	Transitive	Symmetric
disconnected (dc)	✗	✓
externally connected (ec)	✗	✓
partially overlapping (po)	✗	✓
tangentially proper part (tpp)	✗	✗
tangentially proper part inverse (tppi)	✗	✗
non-tangentially proper part (ntpp)	✓	✗
non-tangentially proper part inverse (ntppi)	✓	✗
equal (eq)	✓	✓

**Table 2:** List of temporal relations

Name (abbrev.)	Transitive	Symmetric	Name (abbrev.)	Transitive	Symmetric
before (<)	✓	✗	after (>)	✓	✗
meets (m)	✗	✗	met-by (mi)	✗	✗
overlaps (o)	✗	✗	overlapped-by (oi)	✗	✗
during (d)	✓	✗	contains (di)	✓	✗
starts (s)	✓	✗	started-by (si)	✓	✗
finishes (f)	✓	✗	finished-by (fi)	✓	✗
equal (=)	✓	✓			

#### Definition 2

(Knowledge Graphs) Formally, a Knowledge Graph (KG) can be represented as $G = (V, E)$, where *V* is the set of nodes/entities and *E* is the set of edges with labels, denoting relations held between two entities. A statement then consists of a head entity, a relation, and a tail entity, written as $\langle h, r, t \rangle$, where $h,t \in V$ and $r = \sigma (e), e \in E$. $\sigma$ is a mapping function from an edge to its label. One way to represent such a type of knowledge is known as the RDF (Resource Description Framework), a standard mostly used in the Semantic Web literature. We use the term Knowledge Graph here to denote such a set of RDF statements. Naturally, a statement claiming a spatial or temporal relation between two entities (i.e., geometries or temporal intervals) can be represented as a triple. For instance, a statement that geometry A is *disconnected* to geometry B can be represented as triple $\langle A, \textit{dc}, B \rangle$. Note that we use a unified name – spatial KGs (SKGs) – to refer to KGs involving only spatial relations or/and temporal relations.

#### Definition 3

(Knowledge Graph Embedding) Given their symbolic nature, it is difficult to apply RDF-based knowledge graphs directly to applications that require notions such as quantitative measurements of similarity. For instance, most recommender systems are built upon sub-symbolic approaches and it is hard for symbolic KGs to contribute directly. In order to address this limitation, knowledge graph embeddings (KGE) were proposed, which aim at encoding entities and relations of a KG into a high-dimensional continuous vector space while preserving the underlying structures. Specifically, a KGE model projects symbolic representations of a head entity and a tail entity – *h* and *t*, to points in a continuous vector space – their numeric vector representations, $\textbf {h}$ and $\textbf {t}$, respectively. Additionally, it assumes the relation *r* acts as a transformation operator, transforming $\textbf {h}$ to $\textbf {t}$ in this continuous space, such as translation, rotation, etc. Note that we use plain symbols (e.g., *h*) to denote symbolic representations and the bold format (e.g., $\textbf {h}$) to denote numeric vector representations.

Mathematically, the embedding of an entity, or a relation, is mostly formalized as $\textbf{v} \in {\mathbb{R}^{d}}$, or $\textbf{r} \in {\mathbb{R}^{d}}$, in Euclidean space. Trained on symbolic representations of statements presented in KGs, a KGE model is optimized towards minimizing the loss of reproducing those presented statements. More details on embedding models will be reviewed in Section 3.

#### Definition 4

(Entity Inference) Entity Inference refers to answering queries in which one of the entity in a statement is missing, usually expressed as either $\langle ?h, r, t \rangle$ or $\langle h, r, ?t\rangle$, corresponding to missing head or missing tail entities. A plain text example would be *which city is located in California?*, or *which event occurred during the COVID-19 pandemic?*

#### Definition 5

(Relation Inference) Relation Inference refers to inferring the relation between two entities, usually in the form of $\langle h, ?r, t \rangle$. Example queries include: *what is the topological relation between Los Angeles to California?* and *which temporal relation holds between the Bronze Age and Stone Age?*

#### Definition 6

(Quaternion) A quaternion *q* has the form of $q=a+bi+cj+dk$, where $a, b, c, d \in \mathbb {R}$ and *a* is the real part and *bi*, *cj*, *dk* are three imagery parts. Alternatively, we can express a quaternion as $[a, \textbf {u}]$, where $\textbf{u} \in {\mathbb{R}^{3}}$, consisting of three imagery components. *q* is a pure quaternion when $a=0$.

It was first introduced in 1843 by Irish mathematician William Rowan Hamilton and applied to mechanics in 3D space. We can view it as a generalization of complex numbers (i.e., $a+bi$) but it contains two more imagery parts. Similar to multiplication over complex numbers, there is a rule for the three imagery units *i*, *j*, *k*: $i^2=j^2=k^2=ijk=-1$. According to polynomial multiplication, the multiplication of two quatertions $q_x=a+bi+cj+dk$ and $q_y=e+fi+gj+hk$ can be calculated as below:

1 $$\begin{aligned} q_xq_y = (a+bi+cj+dk)*(e+fi+gj+hk)=\left[ \begin{array}{cccc} a &{} -b &{} -c &{} -d\\ b &{} a &{} -d &{} c \\ c &{} d &{} a &{} -b \\ d &{} -c &{} b &{} a \end{array}\right] \left[ \begin{array}{c} e \\ f \\ g \\ h \end{array}\right] \end{aligned}$$

According to (1), we can easily derive that $q_xq_y \ne q_yq_x$, meaning that quaternion multiplication does not conform to the commutative law. This lays the foundation of modeling asymmetric composition tables for qualitative spatial and temporal reasoning, which will be discussed in Section 4.

Important properties and definitions of quaternions are given as below:

1. *Inversion of a quaternion*: $qq^{-1}=q^{-1}q=1~(q \ne 0)$.
2. *Conjugate of a quaternion*: $q^*=a-bi-cj-dk=a-\textbf {u}$. In addition, $(pq)^*=q^*p^*$.
3. *Norm of a quaternion*: $\Vert q \Vert := \sqrt{qq^*} = \sqrt{q^*q} = \sqrt{a^2 + b^2 + c^2 + d^2} = \sqrt{a^2 + \Vert \textbf {u} \Vert ^2}$. When $\Vert q \Vert =1$, we call *q* a unitary quaternion, denoted as $q_u$.

Because $qq^*=q^*q=\Vert q \Vert ^2$, one way of deriving quaternion inverse is $q^{-1}=\frac{q*}{\Vert q \Vert ^2}$. In particular, when *q* is a unitary quaternion, $q^{-1}=q^*$.

#### Definition 7

(Hyperbolic Space) Hyperbolic space is a homogeneous space which exhibits hyperbolic geometry with a constant negative sectional curvature.

There are different hyperbolic models to describe hyperbolic space mathematically, such as the Poincaré plane model [39] and the hyperboloid model (the Lorentz model)  [40]. Here, we introduce the Poincaré ball model, which is the generalization of the Poincaré plane model. Mathematically, a *d*-dimensional Poincaré ball of radius $\frac{1}{\sqrt{c}}~(c>0)$ can be expressed as ${\mathbb{B}^{d}_{c}} =\{\textbf{x} \in {\mathbb{R}^{d}} : c\Vert \textbf{x} \Vert ^2 < 1\}$, where $\Vert \cdot \Vert$ is the Euclidean norm. Such a ball has a negative curvature $-c$, and with a larger *c*, the space is more curved. Note that Euclidean space has a curvature of zero, corresponding to $c=0$, and spherical space has a constant positive curvature. When $c=1$, the distance between two points in the hyperbolic space is given by:

2 $$\begin{aligned} d_H(\mathbf {x}, \mathbf {y}) = \textit{arcosh}(1+2\frac{\Vert \mathbf {x} - \mathbf {y}\Vert ^2}{(1-\Vert \mathbf {x} \Vert ^2)(1-\Vert \mathbf {y} \Vert ^2)}) \end{aligned}$$

where $\Vert \cdot \Vert$ is the Euclidean norm.

This formula provides a desirable property that allows hyperbolic space to embed trees/hierarchical data. According to this formula, we can observe that when a point is close to the origin (i.e., $\Vert \textbf {x} \Vert \approx 0$), the distance between it and any other point will be smaller. Conversely, as points move towards the boundary of the ball (e.g., $\Vert \textbf {x} \Vert \approx 1$), the distance will be larger and the distance $d_H(\textbf {x}, \textbf {y})$ between two points approaches $d_H(\textbf {x}, 0)$ + $d_H(0, \textbf {y})$. Also, as points move away from the root/origin, more “space” is available to separate points (e.g., nodes in a tree) in hyperbolic space. This is analogous to the shortest distance between two sibling nodes in a tree, which is equal to the length of the path through their parent. This means hyperbolic distance exhibits a desirable resemblance to tree metrics. Figure 3 illustrates how a tree-like 2D embedding space looks like.

**Fig. 2:**
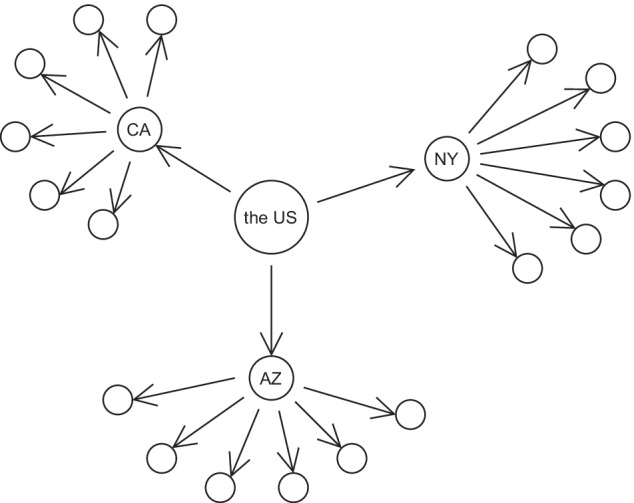
Example of a hierarchical tree. This tree is induced by ntppi (non-tangentially proper part inverse) relation, which means a preceding entity has a nttpi relation to its succeeding entities in this tree

**Fig. 3:**
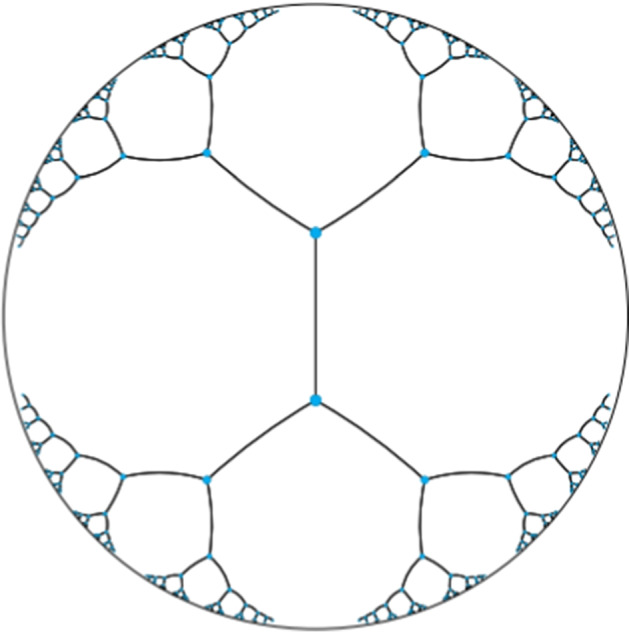
Illustration of embedding a hierarchical tree (with two being the branching factor) into a 2D hyperbolic plane. Distances between any two directly connected points (in blue) are equal and distances grow exponentially when approaching to the edge of the plane. (source from [41])

## Related work

A plethora of Knowledge Graph Embedding (KGE) models have been developed in the past decade. Relations in KGs have different properties, such as symmetry, anti-symmetry, inversion, and transitivity [42]. Different models preserve varying properties due to distinct ways of manipulating relations. Accordingly, we roughly divide them into four groups – translation, rotation, mixed manner, and others. Particularly, this group focuses on which properties of relations (e.g., symmetric and inverse) are preserved and whether the model is able to encode relation composition by design. Last but not least, we review related work on hyperbolic embeddings, which sheds lights on modeling hierarchical relations.

### Relations as translation

The most representative KGE model is TransE [43]. It assumes that for a statement $\langle h, r, t\rangle$, $\textbf {t}$ is resulted from $\textbf {h}$ being translated by $\textbf {r}$ in a vector space. Translation operation in a *real* vector space can be easily achieved by vector addition, and thus the idea of TransE is formalized as $\textbf {h}+\textbf {r} = \textbf {t}$. A number of variants were proposed subsequently to address issues with the original TransE. For example, TransH argued that TransE cannot deal with other types of relations except for 1-to-1 relation type, and, thus, introduced relation-aware hyperplanes [44]. TranSparse introduced adaptive sparse matrices to address the heterogeneity and imbalance issues of entities and relations in KGs [45]. This group of methods is simple yet very effective, and lays the foundation of most KGE methods. However, they fail to encode simple properties of relations and logic patterns. For instance, they cannot model symmetric property of relations. If relation *r* is symmetric, both $\textbf {h}+\textbf {r} = \textbf {t}$ and $\textbf {t}+\textbf {r} = \textbf {h}$ should hold according to TransE, which leads $\textbf {r}$ to be close to $\textbf {0}$. Additionally, although TransE is able to achieve relation composition, the order of relations is not considered. Namely, it presumes that $r1 \circ r2 = r2 \circ r1$. Therefore, TransE ignores the **non-commutativity** law in relation composition, which causes issues in modeling role chains in composition tables for spatial and temporal reasoning. Moreover, TransE cannot deal with **hierarchical relations** either.

### Relations as rotation

One seminal example in this group is RotatE, which assumes that a relation acts as a rotation in 2D space and encodes a relation as a unit complex vector [42]. Similar to TransE in the *real* space, RotatE can be formalized as $\Vert \textbf {h} \otimes \textbf {r} - \textbf {t} \Vert =0$, where $\otimes$ is the vector multiplication in the *complex* space instead. RotatE by design succeeds in modeling multiple logic patterns, such as symmetry, anti-symmetry, inversion, and relation composition. However, it is incapable of dealing with the order of relations in composition, either. Recently, due to the non-commutative law of quaternion multiplication, quaternions, which have two more imaginary elements than complex numbers, have been introduced to address this issue. RotatE3D assumes that a tail entity is resulted from a head entity being rotated by $\textbf {r} in 3D$ [46]. Despite its effectiveness in capturing various logic patterns, it falls short of modeling hierarchical relations from transitive relations. Such relations are in fact prominent in spatial and temporal reasoning since most spatial/temporal relations are transitive. In this paper, we also make use of quaternions to capture additonal logic patterns and extend it to hyperbolic space in order to encode hierarchical structures.

### Relations as mixed operators

Recently,  [47] argue that existing work considers the relation to be either a translation or rotation operator but not both, thus limiting the representational and inferring ability of sub-symbolic models. Hence, they introduce dual quaternions to represent relations, which embrace the properties of translation and rotation simultaneously. Despite its intuitive physical and geometric interpretations, the unified framework do not improve significantly on data sets that encode hierarchical hypernym relations, such as *specific type of*.

### Other methods

Another track of studies are based on tensor factorization, such as DistMult [48] and RESCAL [49] in *real* space and ComplEx [50] and TNTComplEx [51] in *complex* space. This type of methods measures the compatibility score of two entities and a relation in a statement. For example, DistMult defines the score as the result of $\textbf {h} \odot \textbf {r} * \textbf {t}^T$, where $\odot$ is the element-wise vector multiplication and $*$ the dot product. Such methods do not have intuitive geometric interpretations and often fail to capture logic patterns as well as properties of spatial/temporal relations.

### Hyperbolic embeddings

All the aforementioned methods are not effective in modeling hierarchical data, since their embeddings are built in Euclidean space. Recent embedding methods based on hyperbolic geometry exhibit promising results when modeling parsimonious and taxonomic patterns in data, since hyperbolic geometry is natural to model tree-like structures with low distortion  [38, 52–55]. Specifically, as a counterpart to TransE in the hyperbolic space, MuRP, was proposed by [54] to handle hierarchical data in KGs. It achieves remarkable performance with fewer parameters than TransE. However, MuRP faces the same issues as TransE does since they both conform to the translation assumption. In order to encode various logic patterns and to preserve other properties of relations, [56] proposed to combine hyperbolic rotation and reflection with attention. While substantial improvements are observed, this method mainly focuses on anti-symmetric and symmetric relations. On the contrary, our paper aims at taking a broader range of relation properties (e.g., symmetric and anti-symmetric), inverse relation, and relation composition (i.e., role chains in composition tables) into account when designing an embedding model for QSR/QTR.

## HyperQuaternionE

In this section, we first introduce the motivation of the proposed embedding model and then formulate the idea mathematically.

### Motivation

Composition tables, which specify role chains of relations^1^, have been widely used in traditional qualitative spatial and temporal reasoning methods, and are identified as one of the key reasoning techniques [5, 8, 19]. *An embedding method should also be able to automatically find and take full use of such role chains in its inference and reasoning.* One core requirement for such an embedding method is to model *asymmetric* role chains in composition tables; namely $r1 \circ r2 \ne r2 \circ r1$, where $\circ$ denotes the composition operation. For example, if we know geographic entity A is *disconnected* to geographic entity B and B is *tangential proper part* of geographic entity C, the relation of A to C will fall into one of five possible relations, i.e., *dc*, *ec*, *po*, *tpp* or *ntpp* according to the composition table. By contrast, if we first know A is *tangential proper part* of B and B is *disconnected* to C, then the relation of A to C must be *disconnected*. This means the order of relations in role chains matters. In order to take this into account, we use quaternions, an extension of complex numbers, to automatically capture role chains from training data, thanks to the non-commutative law of quaternion multiplication. Additionally, quaternions can be readily used to model varying properties of relations (e.g., symmetric and anti-symmetric relations) and inverse relations, which further contributes to inference and reasoning over spatial and temporal information.

In addition to the need of capturing role chains in composition tables, we notice that 3/8 spatial relations in RCC8, and 9/13 temporal relations in Allen’s temporal intervals [7] are transitive (see Tables 1 and 2). Geometrically, transitive relations usually induce tree-like structures over entities, in which as the depth of a tree increases, the number of child nodes grows exponentially. As shown in Fig. 2, as the root – the US, branches out, more and more child nodes emerge. Also, although some relations (such as *tpp* and *tppi*) are not transitive, they may still induce a tree-like structure over entities to some degree. *Thereby, an embedding method for spatial and temporal reasoning should be built on a suitable embedding space, which is able to encode non-Euclidean structures exhibited in data (e.g., hierarchies)*. Past works have demonstrated that hyperbolic embeddings are more suitable for data exhibiting non-Euclidean geometric properties, such as hierarchy [38]. This is because hyperbolic space can be naturally viewed as a continuous analogy to hierarchical trees in discrete space and it grows exponentially with an increasing radius, which corresponds to an exponential increase in the number of child nodes with increasing tree depth [54]. Therefore, given the abundance of transitive spatial/temporal relations, we embed entities and relations in hyperbolic space rather than Euclidean space.

Despite the aforementioned advantages of quaternions and hyperbolic space, the technical bottleneck of the model design rests on how to harmonize quaternions and hyperbolic space while preserving their respective properties. The transformation of quaternions, which are originally defined in Euclidean space, into a hyperbolic space is not trivial, since quaternion-related vector operations (e.g., vector addition, matrix-vector multiplication, and vector inner product over quaternions) and geometric metrics (e.g., the closed form of distance) in Euclidean space is hard to be generalized to hyperbolic space.

In this paper, we propose a hyperbolic embedding model, called HyperQuaternionE, in which this challenge is tackled. In the following, we will first introduce preliminary concepts and notations, then propose our model, and finally analyze which relation properties and composition patterns our model can preserve.

### Preliminaries

#### Quaternion multiplication and 3D rotation

As mentioned above, one significant advantage of using quaternions in KGE models lies in the ability of quaternions to model asymmetric role chains in composition tables; namely $r1 \circ r2 \ne r2 \circ r1$. This is guaranteed by the non-commutative law of quaternion multiplication (Definition 6). Here, we give a geometrical interpretation by contrasting the role of complex numbers in 2D rotation and that of quaternions in 3D rotation. In 2D space (see RotatE [42]), a 2D rotation can be achieved by the multiplication of a complex number (i.e., a 2D vector to be rotated) and a unitary complex number (i.e., the rotating angle). The rotation direction is either clockwise or counter-clockwise, and the rotation is around the origin. Thus, the order of two consecutive rotations does not make a difference to the resulting vector. That is, the result of rotating a vector by $\theta _1$ first and then by $\theta _2$ is the same as that of rotating the same vector by $\theta _2$ first and then by $\theta _1$; both equal to rotating a vector by an angle of $\theta _1+\theta _2$ at the end. By contrast, quaternions are related to rotations in 3D space, which are originally used in computer graphics [57, 58]. Any point in 3D space in the form of vectors can be expressed as a pure quaternion, and 3D rotation as quaternion multiplication over a pure quaternion (i.e., the point to be rotated) and a unitary quaternion (i.e., the rotation). Unlike rotations in 2D space, where a vector is always rotated around the origin, each 3D rotation specifies a distinct rotating axis and a rotating angle. That is, rotating results are determined by both rotation axes and angles. As such, the result of performing several 3D rotations over a vector consecutively differs from that of performing the same 3D rotations in another order.

Mathematically, 3D rotations can be formalized as (1). We denote the 3D point ($\textbf{v} \in {\mathbb{R}^{3}}$) to be rotated as a pure quaternion $v=[0, \textbf {v}]$, a unitary quaternion $q_u=[cos(\theta ), sin(\theta )\textbf {u}]$ ($\theta$ is the rotating angle and $\textbf {u}$ is the rotating axis) as the rotating vector and the resulting point as $v^{\prime }=[0, \textbf {v}^{\prime }]$ ($\textbf{v}^{\prime }\in {\mathbb{R}^{3}}$).

#### Theorem 1

(Euler-Rodrigues-Hamilton Formula  [59]) Any rotation in 3D space can be derived by quaternion multiplication. The result of rotating a 3D point $\textbf {v}$ by an angle of $\theta$ around a unit axis $\textbf {u}$ (i.e., $q_u$) can be expressed as follows:

3 $$\begin{aligned} v^{\prime } = v_{\Vert } + q_uv_{\bot } = p_uvp_u^{-1}=p_uvp_u^{*} \end{aligned}$$

where $v_{\Vert }$ is the component of $\textbf {v}$ parallel to $\textbf {u}$ and $v_{\bot }$ the component of $\textbf {v}$ perpendicular to $\textbf {u}$. $q_u=p_u^2$ and $p_u=[cos(\frac{\theta }{2}), sin(\frac{\theta }{2})\textbf {u}]$. This theorem can be interpreted as the component of $\textbf {v}$ perpendicular to $\textbf {u}$ is rotated twice by $\frac{\theta }{2}$ around $\textbf {u}$. Proofs to this theorem can be found in [59, 60].

#### Theorem 2

Product of two unit quaternions is still a unit quaternion.

#### Proof

Let *p* and *q* be two arbitrary quaternions. According to Property 2 in Definition 6, $\Vert pq \Vert = \sqrt{pq(pq)^*}=\sqrt{pqq^*p^*}=\sqrt{p(qq^*)p^*}=\sqrt{pp^*}\sqrt{qq^*}=\Vert p \Vert \Vert q \Vert$. Thus when *p* and *q* are unit quaternions; namely $\Vert p \Vert =\Vert q \Vert =1$, $\Vert pq \Vert =1$, i.e., *pq* is a unitary quaternion. This property ensures that a number of consecutive rotations can be replaced by a single rotation, which is fundamental to the modeling of relation composition.

#### Poincaré Ball Model

Similar to [54] and [56], this work uses a *d*-dimensional Poincaré ball model to form the hyperbolic embedding space for embedding tree-like structures ( Definition 7). Reasons for choosing such a model are two-fold. It provides convenient communication between hyperbolic space and Euclidean space via exponential and logarithmic maps [52], thus making it relatively easy to incorporate quaternions rooted in Euclidean space to hyperbolic space. Moreover, it is well-suited for gradient-based optimization methods (see Section 4.2.1).

When *c* is considered, the hyperbolic distance of two points ${\textbf{x}},{\textbf{y}} \in {\mathbb{B}^{d}_{c}}$ is defined as its geodesic distance in the space, which has the desirable property of forming a tree-like embedding space (see Fig. 3). It is formulated as follows:

4 $$\begin{aligned} d^c(\mathbf {x},\mathbf {y})=\frac{2}{\sqrt{c}}arctanh(\sqrt{c}~\Vert (-\mathbf {x}) \oplus _c \mathbf {y}\Vert ) \end{aligned}$$

where $\textit{arctanh}(\cdot )$ denotes the inverse hyperbolic tangent. The *Möbius addition* (i.e., $\oplus _c$) of two points $\textbf{x},\textbf{y} \in {\mathbb{B}^{d}_{c}}$ can be expressed as below:

5 $$\begin{aligned} \mathbf {x} \oplus _c \mathbf {y} = \frac{(1+2c\mathbf {x}^T\mathbf {y}+c \Vert \mathbf {y}\Vert ^2)\mathbf {x} + (1-c\Vert \mathbf {x} \Vert ^2)\mathbf {y}}{1 + 2c\mathbf {x}^T\mathbf {y} + c^2\Vert \mathbf {x} \Vert ^2 \Vert \mathbf {y} \Vert ^2} \end{aligned}$$

where $\Vert \cdot \Vert$ is the Euclidean norm. We can obtain that $\textbf {x} \oplus _c (-\textbf {x})$ = $(-\textbf {x}) \oplus _c \textbf {x}$ = $\textbf {0}$. This property helps model inverse relations in the embedding space.

#### Bridging quaternion and hyperbolic space

##### Exponential map and logarithmic map

As mentioned in Section 4.1, the difficulty of model design lies in how to simultaneously preserve inherent properties from both hyperbolic space and quaternions that are well-studied in Euclidean space. In this paper, instead of directly generalizing möbius transformation as well as Poincaré distance with quaternion entries [61], we adopt a simple strategy by introducing exponential and logarithmic maps [52], which bridges between tangent space (which sits in Euclidean space) and hyperbolic space. By doing so, we can perform quaternion operations in tangent space while measuring hyperbolic distance in hyperbolic space.

For a point $\textbf{x} \in {\mathbb{B}^{d}_{c}}$, its tangent space representation ($\textbf {x}^E$) is defined as a *d*-dimensional vector, which approximates the hyperbolic space $\mathbb {B}^d_c$ around $\textbf {x}$ (origin). The two mappings ($\text {exp}_0^c(\cdot )$ and $\text {log}_0^c(\cdot )$) at the origin have the following closed-form expressions:

6 $$\begin{aligned} \text {exp}_0^c(\mathbf {x}^E)= & {} \text {tanh}(\sqrt{c}\Vert \mathbf {x}^E \Vert )\frac{\mathbf {x}^E}{\sqrt{c}\Vert \mathbf {x}^E \Vert }=\mathbf {x}^H \end{aligned}$$

7 $$\begin{aligned} \text {log}_0^c(\mathbf {x}^H)= & {} \text {arctanh}(\sqrt{c}\Vert \mathbf {x}^H \Vert )\frac{\mathbf {x}^H}{\sqrt{c}\Vert \mathbf {x}^H \Vert }=\mathbf {x}^E \end{aligned}$$

where $\text {exp}_0^c(\cdot )$ maps $\textbf {x}^E$ in the tangent space to $\mathbb {B}^d_c$ and conversely, $\text {log}_0^c(\cdot )$ maps $\textbf {x}^H$ in $\mathbb {B}^d_c$ to the tangent space. Note that we use $\textbf {x}^H$ to denote $\textbf {x}$ in the hyperbolic space while $\textbf {x}^E$ being in Euclidean space.

### Model formulation

The core idea behind the proposed HyperQuaternionE is to encode relations as 3D rotations, and assumes that for a triple $\langle h, r, t\rangle$, the tail entity *t* is the result of the head entity *h* being rotated by relation *r*. This indicates two key steps in our method: rotating the head entity by the relation and measuring the distance between the tail entity and the head entity after being rotated. Despite being similar to the rotation family introduced in Section 3, the main difference is that in our method these two steps are performed in different spaces. The rotating step is performed in the tangent space with the aim to use quaternions in order to capture role chains from data, and the distance measuring step is executed in hyperbolic space so as to form a tree-like embedding space for hierarchical data. Mathematically, for a triple $\langle h, r, t\rangle$ in a KG, these two steps can be formalized as follows. Note that for entities and relations, their embeddings are first randomly initialized, denoted as $\textbf{h}^{E}, \textbf{r}^{E}, \textbf{t}^{E} \in {\mathbb{R}^{d}}$ (d is the dimension), and are learned automatically through training.

In the first step, a 3D rotation on the head entity *h* performed by relation *r* is achieved by Theorem 1. Concretely, head entities are modeled as 3D points to be rotated, and tail entities are modeled as results of head entities being rotated by relations (i.e., 3D rotation). In order to utilize quaternions to implement 3D rotation, we convert real value entries in $\textbf {h}^E$ and $\textbf {r}^E$ into quaternions. Hence each head embedding $\textbf{h}^{E} \in {\mathbb{R}^{d}}$ can be expressed as $\frac{d}{3}$ pure quaternions. Specifically, it can be written as $V^E_h=[h_1, h_2, ..., h_i]^T$, where $h_i=[0, \textbf {h}_{i}]$ is a pure quaternion and $\textbf{h}_{i} \in {\mathbb{R}^{3}}$ ($i \in \{1, 2, ..., \frac{d}{3}\}$) denotes a 3D point. Similarly, each relation is represented by $\frac{d}{3}$ unitary quaternions, whose embedding can be written as $Q^E_r=[q_{r,1}, q_{r,2},...,q_{r,\frac{d}{3}}]^T$, where each $q_{r,i}$ ($i \in \{1, 2, ..., \frac{d}{3}\})$ is a unitary quaternion. According to (3), 3D rotation in the embedding space is given as follows:

8 $$\begin{aligned} \mathbf {Rot}_{hr,4}^E= & {} \text {Rot3D}(\mathbf {h}^E,\mathbf {r}^E)= Q^E_r \circledcirc V^E_h \circledcirc (Q^E_r)^* \end{aligned}$$

9 $$\begin{aligned} \mathbf {Rot}_{hr}^E= & {} concat(\mathbf {Rot}_{hr,4}^E) \end{aligned}$$

where $\circledcirc$ denotes element-wise quaternion multiplication and $(Q^E_r)^*=[h_1^*, h_2^*, ..., h_i^*]^T$ denotes the conjugate of $Q^E_r$. $\textbf {Rot}_{hr,4}^E$ is the rotating result of the head entity and contains $\frac{d}{3}$ pure quaternions. $concat(\cdot )$ is to concatenate three imagery components of these pure quaternions in order to recover the original dimension *d*.

In the second step, to form a tree-like embedding space for hierarchical data, we measure the distance between the resulting head embedding and the tail embedding in hyperbolic space. Since the first step is performed in tangent spaces, we first map Euclidean embeddings into hyperbolic embeddings via exponential maps shown in (6). However, rather than using a generic curvature *c*, a relation-aware learnable curvature $c_r$ is introduced for each relation because relations of different kinds may yield hierarchical structures of varying degrees. For example, a graph where only the relation *tangential proper part* holds between entities would have a higher hierarchy index than the one induced by the relation *disconnected*. The relation-aware exponential maps are shown below.

10 $$\begin{aligned} \mathbf {Rot}_{hr}^H= & {} \text {exp}_0^{c_r}(\mathbf {Rot}_{hr}^E) = \text {tanh}(\sqrt{c_r}\Vert \mathbf {Rot}_{hr}^E \Vert )\frac{\mathbf {Rot}_{hr}^E}{\sqrt{c_r}\Vert \mathbf {Rot}_{hr}^E \Vert }\end{aligned}$$

11 $$\begin{aligned} \mathbf {t}^H= & {} \text {exp}_0^{c_r}(\mathbf {t}^E) = \text {tanh}(\sqrt{c_r}\Vert \mathbf {t}^E \Vert )\frac{\mathbf {t}^E}{\sqrt{c_r}\Vert \mathbf {t}^E \Vert } \end{aligned}$$

where $\textbf {Rot}_{hr}^H$ and $\textbf {t}^H$ are embeddings of $\textbf {Rot}_{hr}^E$ and $\textbf {t}^E$ in hyperbolic space, respectively.

Finally, the distance is calculated by using the following formula:

12 $$\begin{aligned} d^{c_r}(\mathbf {Rot}_{hr}^H,\mathbf {t}^H)=\frac{2}{\sqrt{c_r}}arctanh(\sqrt{c_r}~\Vert (-\mathbf {Rot}_{hr}^H) \oplus _{c_r} \mathbf {t}^H \Vert ) \end{aligned}$$

Equation (12) is originated from (4), but contains a relation-aware learnable curvature $c_r$ to consider the difference of embedding spaces induced by various relations.

Similar to previous work [42, 48], we optimize the model by minimizing the distance between $\textbf {Rot}_{hr}^H$ and a valid tail *t* (meaning that $\langle h, r, t\rangle$ exists in our KG) and maximizing that to a negative tail. More specifically, for a triple $\langle h, r, t\rangle$ in a KG, *t* itself is a positive tail and we construct negative tails by replacing *t* with another entity (i.e., $t^\prime$), which is randomly picked from all other entities. It is done by *n* times in order to obtain *n* negative tails. Finally, the optimizer is to pull the correct *t* towards $\textbf {Rot}_{hr}^H$ as close as possible while pushing negative ones far away, which can be formalized as:

13 $$\begin{aligned} \mathcal {L} = -log \; \sigma (\gamma - d^{c_r}(\mathbf {Rot}_{hr}^H,\mathbf {t}^H)) - \frac{1}{n}\sum _{i=1}^{n}log \; \sigma (d^{c_r}(\mathbf {Rot}_{hr}^H,\mathbf {t^\prime _i}^H)) - \gamma ) \end{aligned}$$

where $\sigma$ denotes the sigmoid function and $\gamma$ is a hyper-parameter indicating the tolerance of distance between the positive/negative and the resulting entity embedding.

Likewise, with regard to relation inference, for each positive triple $\langle h, r, t\rangle$, we corrupt it by replacing *r* with other (spatial/temporal) relations $n_r$ times so as to generate $n_r$ relation-based negative samples. To consider both tasks, we construct a joint loss function and use a scalar $\beta$ to adjust their respective contributions:

14 $$\begin{aligned} \mathcal {L}^\prime = \mathcal {L} - \beta \frac{1}{n_r}\sum _{i=1}^{n_r}log \; \sigma (d^{c_{r_i}}( {Rot}_{hr_i}^H, {t}^H)) - \gamma ) \end{aligned}$$

Last but not least, we introduce a way of representing relations such that they can be ensured to be unitary quaternions. This is of great importance to achieve 3D rotations based on Theorem 1. Recall that only three values are needed to determine a unitary quaternion. So for any three arbitrary values $\alpha , \theta _1, \theta _2 \in [-\pi , \pi ]$, a unitary quaternion can be constructed as follows:

15 $$\begin{aligned} q_u=cos(\alpha ) + sin(\alpha )cos(\theta _1)cos(\theta _2)i + sin(\alpha )cos(\theta _1)sin(\theta _2)j+ sin(\alpha )sin(\theta _1)k ~ \end{aligned}$$

Based on the definition of quaternion norm (see Property 3), $\Vert q_u \Vert =1$ can be readily ensured (See Appendix 1 for proofs). In what follows, we analyze relation properties and composition patterns that are preserved by using the proposed model.

#### Lemma 1

HyperQuaternionE can model symmetric/anti-symmetric properties of relations.

16 $$\begin{aligned} \mathbf {t}_i^E= & {} q_{r,i} \mathbf {h}_i^E q_{r,i}^*\end{aligned}$$

17 $$\begin{aligned} \mathbf {h}_i^E= & {} q_{r,i} \mathbf {t}_i^E q_{r,i}^* \end{aligned}$$

Thus, when we plug (16) into (17), it yields:

18 $$\begin{aligned} \mathbf {h}_i^E = q_{r,i} (q_{r,i} \mathbf {h}_i^E q_{r,i}^*) q_{r,i}^*= q_{r,i}^2 \mathbf {h}_i^E (q_{r,i}^*)^2 \end{aligned}$$

The correspondence of $\textbf {h}_i^E$ in hyperbolic space is given by (6):

19 $$\begin{aligned} \mathbf {h}_i^H = \text {tanh}(\sqrt{c_{r}}\Vert \mathbf {h}_i^E \Vert )\frac{\mathbf {h}_i^E}{\sqrt{c_{r}}\Vert \mathbf {h}_i^E \Vert } \end{aligned}$$

When we substitute $\textbf {h}_i^E$ in (19) with (18), we obtain the following:

$$\begin{aligned} \mathbf {h}_i^H= & {} \text {tanh}(\sqrt{c_r}\Vert \mathbf {h}_i^E \Vert )\frac{q_{r,i}^2 \mathbf {h}_i^E (q_{r,i}^*)^2}{\sqrt{c_r}\Vert \mathbf {h}_i^E \Vert } \\= & {} q_{r,i}^2 \frac{\text {tanh}(\sqrt{c_r}\Vert \mathbf {h}_i^E \Vert ) \mathbf {h}_i^E}{\sqrt{c_r}\Vert \mathbf {h}_i^E \Vert } (q_{r,i}^*)^2 \\= & {} q_{r,i}^2 \mathbf {h}_i^H (q_{r,i}^*)^2 \\\Leftrightarrow & {} q_{r,i}^2 = \pm 1 \end{aligned}$$

It indicates that the sufficient and necessary condition of modeling symmetric relations is that $q_{r,i}^2 = \pm 1$ holds. Clearly, in 3D space, a rotation angle of $k*180^\circ$ ($k \in \{1,3,5,...\}$) satisfies this condition. Likewise, we can derive that $q_{r,i}^2 \ne \pm 1$ is the sufficient and necessary condition for modeling anti-symmetric relations.

#### Lemma 2

HyperQuaternionE can model inversion of relations.

If $\langle h, r1, t\rangle$ and $\langle t, r2, h\rangle$ hold, similarly, according to Theorem 1, in the tangent space for each rotation we have:

20 $$\begin{aligned} \mathbf {t}_i^E= & {} q_{r1,i} \mathbf {h}_i^E q_{r1,i}^*\end{aligned}$$

21 $$\begin{aligned} \mathbf {h}_i^E= & {} q_{r2,i} \mathbf {t}_i^E q_{r2,i}^* \end{aligned}$$

The correspondence of $\textbf {h}_i^E$ in hyperbolic space is given by (6):

22 $$\begin{aligned} \mathbf {h}_i^H = \text {tanh}(\sqrt{c_{r2}}\Vert \mathbf {h}_i^E \Vert )\frac{\mathbf {h}_i^E}{\sqrt{c_{r2}}\Vert \mathbf {h}_i^E \Vert } \end{aligned}$$

Then, we can obtain:

$$\begin{aligned} \mathbf {h}_i^H= & {} \text {tanh}(\sqrt{c_{r2}}\Vert \mathbf {h}_i^E \Vert )\frac{(q_{r2,i}q_{r1,i}) \mathbf {h}_i^E (q_{r2,i}q_{r1,i})^*}{\sqrt{c_{r2}}\Vert \mathbf {h}_i^E \Vert } \\= & {} (q_{r2,i}q_{r1,i}) \frac{\text {tanh}(\sqrt{c_{r2}}\Vert \mathbf {h}_i^E \Vert ) \mathbf {h}_i^E}{\sqrt{c_{r2}}\Vert \mathbf {h}_i^E \Vert } (q_{r2,i}q_{r1,i})^* \\= & {} (q_{r2,i}q_{r1,i}) \mathbf {h}_i^H (q_{r2,i}q_{r1,i})^* \\\Rightarrow & {} q_{r2,i} = \pm q_{r1,i}^* \end{aligned}$$

Clearly, this equation can have multiple solutions. For instance, for a relation *r*1 with its quaternion representation in a dimension being $q_{r1,i}=[\alpha _1, \textbf {v}_1]$, it inverse relation *r*2 at the same dimension can be constructed as $q_{r2,i}=[\alpha _1, -\textbf {v}_1]$ or $q_{r2,i}=[-\alpha _1, \textbf {v}_1]$.

#### Lemma 3

HyperQuaternionE can capture non-commutative patterns of relation composition. In special cases, HyperQuaternionE can model commutative patterns.

Non-commutative composition of relations implies that $r1 \circ r2 \ne r2 \circ r1$ while commutative composition indicates that $r1 \circ r2 = r2 \circ r1$. Here $\circ$ refers to quaternion multiplication. According to Theorem 2, $r1 \circ r2$ yields another relation *r*3, namely $r1 \circ r2=r3$, and likewise $r2 \circ r1=r4$. Due to the non-commutative law of quaternion multiplication (see (1)), $r3 \ne r4$ can be naturally guaranteed. On the other hand, in special cases, for example, when *r*1 and *r*2 share the same rotating axis, we can conclude that $r1 \circ r2 = r2 \circ r1=r3=r4$ (i.e., commutative composition).

Table 3 summarizes varying properties of relations and patterns of relation composition that different models can preserve. As can be seen, the proposed HyperQuaternionE achieves all. Note that our HyperQuaternionE method can be applied to other KGs where aforementioned properties of relations and patterns of relation composition exist commonly. We leave the investigation as future work.

**Table 3:** Varying properties and patterns modeled by differing models

		TransE [43]	RotatE [42]	Rotate3D [46]	HyperRotatE [56]	HyperQuaternionE
Property	Symmetric	✗	✓	✓	✓	✓
	Anti-symmetric	✓	✓	✓	✓	✓
	Inversion	✓	✓	✓	−	✓
Composition	commutative	✓	✓	✓	−	✓
	non-commutative	✗	✗	✓	−	✓
Hierarchy	induced by transitive relations	✗	✗	✗	✓	✓

*Note that - means inapplicable

## Experiments

In this section, we introduce the experimental data and baseline methods. Plus, experimental results are reported quantitatively and qualitatively.

### Data preparation

We synthesize two datasets –region187 and interval205 for spatial reasoning and temporal reasoning, respectively. Both datasets are generated from randomly generated rectangular regions and intervals. For region187, we first generate 200 pairs of points. Each pair is used to represent the top left and bottom right corners of a rectangle. We further filter out invalid cases (e.g., the top left and the bottom right points share the same x/y value). Then we calculate the spatial (topological) relation between any two rectangles based on their geometries and organized them as triples (e.g., (rectangle 1, *dc*, rectangle 2)). Additionally, we sample 5 rectangles to establish more *eq* relations since it is relatively rare to yield the same rectangles from the previous step. A similar process is adopted to generate interval205. Finally, we randomly split both datasets into training ($70\%$), validation($15\%$), and testing sets ($15\%$). Table 4 describes statistics of the two datasets.

**Table 4:** Statistics of region187 and interval205

Dataset	#entities	#relations	#train	#valid	#test
region187	187	8	24,460	5,241	5,243
interval205	205	13	29,399	6300	6301

### Baseline methods

Our model is compared with four baselines: three embedding models and one traditional method used in spatial and temporal reasoning. The three embedding methods (i.e., RotatE, QuaternionE/Rotate3D, HyperRotatE/RotH) are chose upon Table 3,^2^. All these models are unified in the same framework and thus adopt the same protocols for data processing, training, as well as evaluation.

Traditional methods are built upon path consistency checking over a constraint network, where nodes represent entities (e.g., rectangles or intervals in this paper) and edges are labelled with a set of possible relations between entities [7]. By propagating temporal/spatial composition tables over the network [62], this network will be refined as the relations between entities that do not conform to composition tables will be ruled out. Similarly, in our experiment, we construct a network by using training and testing datasets, where relations in the testing set all are changed to be a set of all possible relations in the beginning (namely eight relations for spatial reasoning and thirteen relations for temporal reasoning). Through propagation, relations that lead to inconsistency will be discarded and the remaining relations are viewed as inference results. Figure 1 gives an illustrative interpretation. We name this method as constraint network method and use an open-sourced package to implement it^3^.

### Experimental settings

In order to achieve a fair comparison, we ensure that all compared models share approximately the same number of parameters. The number of learnable parameters used in each model is shown in Appendix 2. Similar to [56], we carry out two experimental settings – low-dimensional and high-dimensional. More details on the number of parameters as well as the best parameter setting are shown in Table 5. Note that four hyper-parameters are chose from various ranges: learning rate – *lr*:[0.05, 0.1], margin in (14) – $\gamma$:[8, 10, 12], batch size – *b* :[512, 1024] and negative samples – *n*: [8, 16, 32, 64]. For the weighting parameter $\beta$ in (14), we set it as 0.5 empirically.

**Table 5:** Best parameter setting for each model on two datasets (low-dimensional vs. high-dimensional)

Models on region187	low-dimensional	high-dimensional
HyperQuaternionE	lr0.1-b512-g8-n8-h30 (5,858)	lr0.05-b1024-g12-n8-h120 (23,408)
HyperRotatE	lr0.1-b512-g0-n8-h26 (5,681)	lr0.05-b1024-g0-n64-d110 (23,405)
QuaternionE	lr0.1-b1024-g12-n8-h30 (5,850)	lr0.1-b1024-g12-n64-h120 (23,400)
RotatE	lr0.1-b512-g10-n64-h16 (6,112)	lr0.1-b1024-g12-n64-h62 (23,684)
Models on interval205	low-dimensional	high-dimensional
HyperQuaternionE	lr0.01-b1024-g8-n8-h45 (9,823)	lr0.05-b1024-g8-n32-h150 (32,713)
HyperRotatE	lr0.05-b1024-g0-n16-h40 (9,978)	lr0.05-b1024-g0-n64-h132 (32,631)
QuaternionE	lr0.1-b1024-g12-n16-h45 (9,810)	lr0.1-b512-g12-n64-h150 (32,700)
RotatE	lr0.05-b1024-g12-n32-h23 (9,729)	lr0.05-b1024-g12-n32-h78 (32,994)

#### Evaluation metrics

At testing, we compare different methods on two tasks: entity inference (Definition 4) and relation inference (Definition 5). Note that the constraint network method can only achieve the relation inference task while being incapable of inferring missing entities. Specifically, for each test sample $\langle h, r, t\rangle$, we generate three queries: $\langle ?h, r, t\rangle$ and $\langle h, r, ?t \rangle$ for the former task, and $\langle h, ?r, t\rangle$ for the latter. For each query, we utilize (12) as the scoring function and measure distances between each candidate entity or relation and the correct answer. Then all candidate entities/relations are scored and later ranked by distances in the inference process. A smaller distance means a better fit to a query, indicating a higher likelihood of the entity/relation to be true. Following previous works [43, 50, 63], we choose two popular ranking-based metrics, namely Mean Reciprocal Rank (*MRR*), which measures inverse ranks of gold answers over all test samples on average and *H*@*k* ($k \in \{1, 2, 3\}$), which measures the proportion of gold answers being ranked in the top *k* on average. In general, the higher the rank is, the better a model performs. Meanwhile, during the evaluation, we also follow [43] to filter out inference results that are already true in the KG^4^.

### Experimental results

In this subsection, we first report the performance of our model in comparison with other embedding methods and traditional methods, and analyze what our model learns.

#### Comparison with embedding methods

**Fig. 4:**
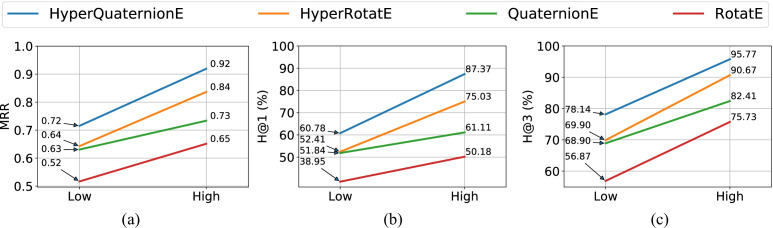
Model performance on the region187 dataset – entity inference task

**Fig. 5:**
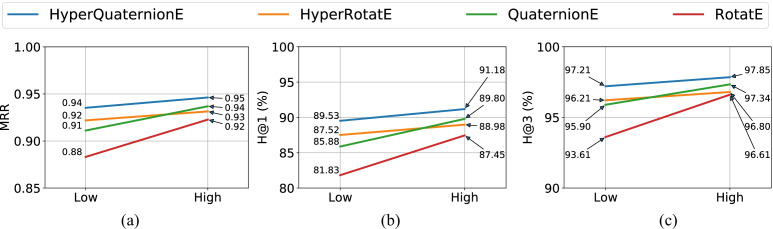
Model performance on the temporal205 dataset – entity inference task

**Fig. 6:**
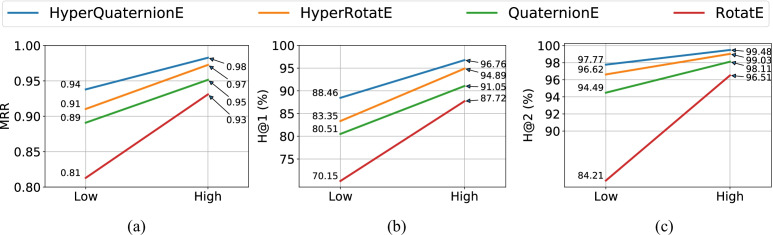
Model performance on the region187 dataset – relation inference task

**Fig. 7:**
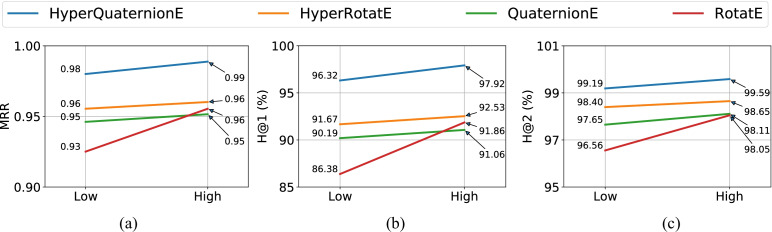
Model performance on the interval205 dataset – relation inference task

Figures 4 and 5 show our model performance against baseline embedding methods on the task of entity inference, and Figs. 6 and 7 report results on the task of relation inference. We summarize our main findings as below.

(1) **Our proposed method consistently outperforms baseline methods on two datasets in both low-dimensional and high-dimensional settings.** More specifically, in terms of the task of entity inference, compared with the strongest baseline method - HyperRotatE (in orange), HyperQuaternionE (in blue) gains around 8-point improvements in terms of *MRR* in both low-dimensional and high-dimensional settings, respectively (see Fig. 4). In terms of *H@1*, HyperQuaternionE beats HyperRotatE by around 8% in the low-dimensional setting, and by around 12% in the high-dimensional setting. On the interval205 dataset (See Fig. 5), all embedding methods perform very well and the difference between our method and HyperRotatE is slightly subtle. Specifically, even in the low-dimensional setting (with 9,823 parameters), HyperQuaternionE reaches to around $91\%$ in terms of *H@1* and $97.85\%$ in terms of *H*@3.

In terms of the relation inference task (see Fig. 6 and 7), HyperQuaternionE still consistently outperforms all other embedding methods on all evaluation metrics. For example, HyperQuaternionE surpasses HyperRotatE by around $5\%$ and 3 points in terms of *H@1* and *MRR* on the interval205 dataset, respectively. On the region187 dataset, our method improves HyperRotatE by around $5\%$ and $2\%$ in terms of *H@1* in the low-dimensional setting and high-dimensional setting, respectively. It is worth-noting that all embedding methods perform very well on the task of relation inference with *H@1* being over $95\%$. We compare our method with traditional reasoning methods in Section 5.4.3 on this task.

(2) **Hyperbolic embedding methods are more robust than Euclidean methods when handling spatial and temporal reasoning**. Apparently, hyperbolic embedding methods (i.e., HyperQuaternionE consistently exceeds their Euclidean alternatives (i.e., QuaternionE and RotatE) on both datasets for both tasks. For example, in the high-dimensional setting in Fig. 4a, HyperQuaternionE improves over QuaternionE by around 14 points and HyperRotatE gains around 19 points against RotatE. In Fig. 5b, HyperQuaternionE and HyperRotatE achieve improvements of $6.6\%$ and $6\%$ over their Euclidean alternatives, respectively. More remarkably, we find that the performance of hyperbolic embedding methods in low-dimensional settings is even comparable to that of their Euclidean equivalents in high-dimensional settings. In Fig. 4a and b, HyperQuaternionE in the low-dimensional setting (5,858 parameters) is on a par with QuaternionE in the high-dimensional setting (23,400 parameters). For instance, the difference in *MRR* (0.72 for low-dimensional HyperQuaternionE v.s. 0.73 for high-dimensional QuaternionE) is subtle.

#### Comparison with traditional reasoners

We compare embedding methods in high-dimensional settings with a traditional method (i.e, the constraint network method which relies on composition tables) on the relation inference task. A challenge in this experiment is how to evaluate their inference results quantitatively. A traditional reasoner built upon RCC8/temporal composition tables usually yields *a set of* possible relations that could be held between two entities, despite the fact that there must be exactly one (spatial/temporal) relation holds between two entities. Differently, embedding methods usually output *a ranked list* of relations sorted by a scoring function (e.g., (12)); see Table 6 for more details. In order to compare these two methods, we use five evaluation metrics - two absolute metrics for accuracy evaluation, two relative metrics for error evaluation and one for recall evaluation.

In terms of absolute metrics, we stick with *H@1* and *MRR* to evaluate their inference accuracy. For *H@1*, when the constraint network method yields only one relation, we call it a success since in theory only one (spatial/temporal) relation would be held between any two entities; otherwise, we view it as a “failure”. This is a “strict” evaluation. In order to take into account the contribution of those “failures”, we use *MRR*. In this case, if the constraint network method yields exactly one relation for a testing sample (e.g., $\langle h, ?r, t\rangle$), then the score for this sample is 1. Otherwise, the score for a sample with a set of inferred relations will be the average *MRR*s of the correct relation being ranked at any position in the answer set, which is $\frac{1}{\vert s \vert }\sum _{n=1}^{\vert s \vert } \frac{1}{n}$ ($\vert s \vert$ is the number of elements in the set *s*).

**Table 6:** Examples of relation inference results. Both methods aim to infer the relation between a subject and an object. Column Relation denotes the correct relation, column Constraint Network and HyperQuaternionE denote their respective inference results. Note that constraint network method outputs a set of possible relations while HyperQuaternionE yields a ranked list of relations. Here we only show Top 1 relation from the ranked list

Examples	Subject	Relation	Object	Constraint Network	HyperQuaternionE (Top 1)
0	103	*dc*	72	*dc*, *ec*, *po*	*dc*
1	39	*ec*	153	*ec*, *po*	*ec*
2	134	*po*	140	*po*	*po*
3	49	*po*	61	*po*	*po*
4	76	*dc*	92	*dc*	*dc*
5	102	*eq*	186	*dc*, *ec*, *eq*, *po*, *tpp*, *tppi*	*eq*
6	150	*tppi*	31	*po*, *tppi*	*tppi*
7	22	*po*	3	*dc*, *ec*, *po*	*po*
8	65	*tpp*	150	*ntpp*, *tpp*	*tpp*
9	122	*tppi*	40	*po*, *tppi*	*tppi*

**Table 7:** *H@1* and *MRR* on the region187 dataset. ± indicates the following is the standard deviation

	constraint network		HyperQuaternionE	
Training Size	H@1	MRR	H@1	MRR
70%	76.8%	0.927	**96.8%±0.3%**	**0.983±0.002**
60%	74.9%	0.920	**93.5%±0.1%**	**0.965±0.004**
50%	71.3%	0.906	**91.0%±0.5%**	**0.951±0.003**
40%	67.1%	0.890	**88.3%±0.8%**	**0.935±0.003**
30%	60.9%	0.865	**82.8%±0.4%**	**0.902±0.002**

Bold entries indicate the best results in each corresponding comparison

**Table 8:** *H@1* and *MRR* on the interval205 dataset

	constraint network		HyperQuaternionE	
Training Size	H@1	MRR	H@1	MRR
70%	96.8%	0.989	**97.9%±0.2%**	0.989±0.001
60%	96.6%	0.986	**97.1%±0.3%**	0.984±0.002
50%	96.0%	0.984	**96.7%±0.2%**	0.982±0.002
40%	95.0%	0.981	**95.8%±0.3%**	0.979±0.004
30%	93.0%	0.971	**94.2%±0.4%**	0.970±0.002

Bold entries indicate the best results in each corresponding comparison

Table 7 and 8 show the accuracy comparison between the constraint network method and HyperQuaternionE with varying sizes of training data. We find that **our model outperforms the constraint network method on spatial reasoning tasks by significant margins in terms of different training sizes and achieves comparable results on temporal reasoning tasks**. With respect to the “strict” accuracy evaluation – *H@1*, HyperQuaternionE consistently surpasses the constraint network method on both spatial and temporal relation inference. In Table 7, HyperQuaternionE beats the constraint network method by over 20% for all different training sizes on the region187 dataset. On the interval205 dataset (see Table 8), our method consistently outperforms the constraint network method by around $1\%$. Additionally, with the training size increasing, we observe that both methods improve as we expect. It is worth-noting that even with only $30\%$ data (of the entire graph) being in the training set, our method can obtain $82.8\%$ and $97.9\%$ in terms of *H@1* on these two datasets, respectively. In terms of *MRR*, a similar pattern of their performance is observed: HyperQuaternionE outperforms the constraint network method by around 5 points on the region187 dataset; however the differences between both methods on the interval205 dataset are relatively subtle but both achieve very high scores (i.e., over 0.97) for all different training sizes.

Despite the fact that the constraint network method does not necessarily to uncover the single (*true*) relation between entities, inference results are theoretically guaranteed by composition tables based on the amount of data given. Put differently, the correct relation is always a member of the result/answer set. We denote this inferred results as *theoretical results*. Here we are interested in evaluating errors of our inference against the theoretical results. We use two relative metrics - *Error Ratio* and *Recall-Coverage Ratio* to achieve this. *Error Ratio* - *ER* measures the failure of our model against the inference of composition tables. For a testing sample, it examines whether the Top 1 relation produced by our method is a member of the theoretical results yielded by the constraint network method. We use the average score over all testing samples as its final *Error Ratio* of our model. It can be expressed as follows.

23 $$\begin{aligned} Error\, Ratio =\frac{1}{n} \sum \limits _{i=1}^n TrueOrFalse_i \end{aligned}$$

Here, for a testing sample *i*, if Top 1 relation in our ranked list is *not* a member of its corresponding theoretical relation set, then $TrueOrFalse_i$ will be 1; otherwise, $TrueOrFalse_i$ will be 0. *n* is the number of testing samples.

In addition, we introduce a *Recall-Coverage Ratio* - *RC-R* to measure the difficulty of our model in recalling results from the classical RRC8 reasoner. Specifically, for a ranked list of relations produced by our model regarding a testing sample $\langle h, ?r, t \rangle$, we calculate the ratio of the cardinality of the theoretical result set over the minimal length of a ranked list (staring from the first position) containing all relations in the theoretical set. This measure can be formulated as follows:

24 $$\begin{aligned} Recall-Coverage\, Ratio =\frac{1}{n} \sum \limits _{i=1}^n\frac{\vert s_i \vert }{\max _{r \in s_i}pos(r)} \end{aligned}$$

Here, $s_i$ is the result set from the classical RCC8 reasoner for a testing sample *i* and *pos*(*r*) denotes the position index of relation *r* (from $s_i$) in our ranked list (1-index).

Additionally, we calculate the *Recall (R)* of our method. In the literature, *Recall* is defined to measure whether a true relation is contained in the result produced by a model. For the constraint network method, its *Recall* is always 1. As mentioned above, for a testing sample, its inference result always contains the correct relation, since the method performs a filtering-out operation, which excludes impossible relations between two entities. In our method, we also examine our *Recall* against the constraint network method. For each testing sample, we check whether the correct relation is contained in the top $\vert s \vert$ of our ranked list ($\vert s \vert$ is the cardinality of the relation set *s* produced by the constraint network method). This ensures that the sublist of our ranked list used in the *Recall* calculation has the same length as the relation set from the constraint network.

**Table 9:** Error Ratio, Recall-Coverage Ratio and Recall on two datasets

	region187			interval205		
Training Size	RC-R	ER	R	RC-R	ER	R
70%	96.64%	2.29%	100%	98.91%	1.38%	100%
60%	93.80%	4.08%	100%	98.59%	1.83%	100%
50%	92.46%	5.95%	100%	98.38%	1.95%	100%
40%	91.52%	6.69%	100%	97.66%	2.44%	100%
30%	88.68%	9.63%	100%	96.37%	3.49%	100%

Table 9 shows *Error Ratio (ER)*, *Recall-Coverage Ratio (RC-R)* and *Recall (R)* of our method against the theoretical results. As expected, *Error Ratio* increases and *Recall-Coverage Ratio* drops as the training size decreases. When the training size is $70\%$, *ER* is as low as $2.29\%$ and $1.38\%$ on the region187 dataset and interval205, respectively. Meanwhile, *Recall-Coverage Ratio* reaches to $96.64\%$ and $98.91\%$, respectively. Even when the training size drops to $30\%$, *Error Ratio* is still low ($9.63\%$ on the region187 dataset and $3.49\%$ on the interval205 dataset). Similarly, the *Recall-Coverage Ratio* is $88.86\%$ and $96.37\%$, respectively. Moreover, it is worth noting that we achieve the same *Recall* as the constraint network method does, meaning that the correct answer is also contained in the top $\vert s \vert$ of our ranked list. Overall, the results from Table 9 clearly show the suitability of our method for inference.

Summing up all presented evaluations, the results demonstrate that our embedding method can produce results of a *higher* accuracy for reasoning over relations than the constraint network method. Moreover, although our method can also achieve a *Recall* of as high as $100\%$ as the constraint network method does, *Recall-Coverage Ratio* in Table 9 indicates these two methods may adopt different reasoning mechanisms or our embedding method may use other implicit inference. It would be interesting to study and analyze the underlying reasoning techniques in the future. In Section 5.4.4, we qualitatively analyze our model and examine what has been learned by our model from data.

#### Comparison between spatial reasoning and temporal reasoning tasks

By contrasting the performance of spatial reasoning and temporal reasoning (e.g., Figs. 4a and 5a, 6a and 7a, 4b and 5b, etc.), we can easily find that achieving temporal reasoning is relatively easier than spatial reasoning, at least when the proportion of missing relations is the same. Note that we use $70\%$ of the entire dataset as the training set for both spatial and temporal reasoning (see Table 4). In low-dimensional settings (see Figs. 4a and 5a), HyperQuaternionE yields an *MRR* of 0.72 on the region187 dataset while obtaining an *MRR* of 0.94 on the interval205 dataset. Similarly, in Fig. 7b and  6b, HyperQuaternionE in low-dimensional settings yields $88.46\%$ and $96.32\%$ on the region187 dataset and interval205, respectively. Moreover, we observe a similar pattern from Tables 7 and 8. For instance, we can see that when the training size is the same, both the constraint network method and our method are better at reasoning about temporal relations.

**Fig. 8:**
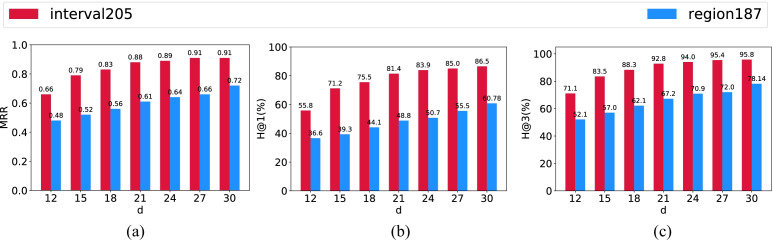
Performance comparison between temporal and spatial entity inference tasks. *d* is the hidden dimension

**Fig. 9:**
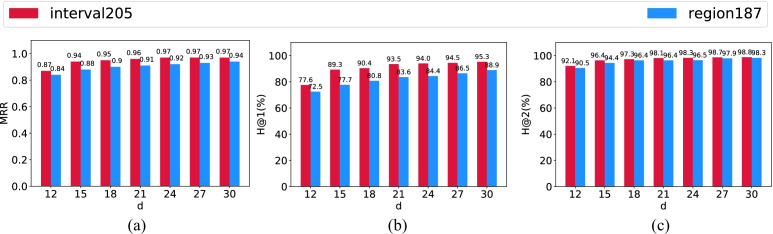
Performance comparison between temporal and spatial relation inference tasks

In order to further test the hypothesis that temporal reasoning is relatively easier to achieve, we conduct experiments to compare the performance of our model in spatial reasoning and temporal reasoning tasks with changing hidden dimensions, which determines the number of learnable parameters (see Appendix 2) and thus impacts the training efficiency^5^. Figures 8 and 9 demonstrate that our model indeed consistently performs better on temporal reasoning tasks, particularly on the task of entity inference. For instance, with a hidden dimension of 12, our model can yield an *H@1* of 55.8 for temporal entity inference while obtaining 36.6 for spatial entity inference. With the hidden dimension increasing, the gap between them is shrinking even though it is still significant. With a hidden dimension of 30, when the model reaches to 0.91 in terms of *MRR* on the temporal entity inference task, *MRR* of the spatial case yields 0.72. This observation may also be viewed as a potential advantage of embedding methods against traditional methods that rely on path-consistency checking (e.g., the constraint network method). For path-consistency checking based methods, as the number of relations increases, composition tables often become more complicated and thus reasoning over relations will be inefficient. That is, the efficiency of the traditional reasoner is bounded by the complication of composition tables as relations involved increase. However, empirical experiments shown above disclose that embedding-based methods like HyperQuaternionE, with less parameters can obtain a even better result when reasoning over temporal relations than over spatial relations; thus they are more efficient on reasoning over temporal relations. This observation indicates the fact that the performance and training efficiency of embedding methods may not be bounded by the complication of composition tables, which is another advantage of embedding methods. We leave more in-depth theoretical and empirical analyses as future work.

#### Qualitative analysis

In this section, we are interested in the question whether embedding methods are able to implicitly learn knowledge from data. This perspective not only suggests promoting embedding methods as a new tool for knowledge discovery, but may also help the design of new models. That is, if some domain knowledge can be learned implicitly, there is no need to make theories/domain knowledge explicit during the model design.

**Fig. 10:**
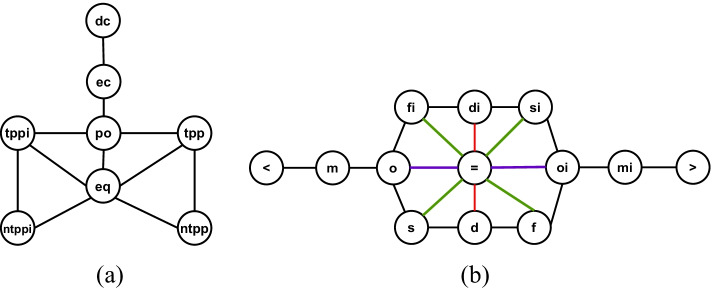
Conceptual neighborhood structure (CNS)  [9, 64]. Figure 10a illustrates conceptual neighbors of spatial relations. Figure 10b reveals conceptual neighbors of temporal relations, in which there are three types of neighboring relations to the relation *equal* (i.e., $=$), distinguished by three different colors

In particular, we examine whether embedding methods could learn conceptual neighborhood structures implicitly, which is fundamental to spatial and temporal reasoning. According to [19, 20, 64], if two relations between pairs of entities (i.e., geometries or events) can be directly transformed from one to the other by continuous deformation of entities (i.e., enlarging, shrinking, lengthening or shortening), these two relations are conceptual neighbors. Conceptual neighborhood structures of spatial and temporal relations are illustrated in Fig. 10.

In order to investigate whether embedding methods manage to learn these structures, we create (spatial/temporal) relation networks. In a spatial/temporal network, nodes are relations and the linkages between relations are determined by the result of the relation inference task. More specifically, in the relation inference task, for a testing sample, (e.g., $<h, ?r, t>$), our model will output a ranked list of all relations sorted by scores in a descent order, in which a relation with a high score means a higher likelihood to be the relation held between *h* and *t*. We pick Top 1 and Top 2 relation from the ranked list and establish a directed edge from Top 1 relation to Top 2 relation to indicate these two relations are likely to be concept neighbors. The underlying rationale is that relations that are conceptual neighbors are hard to be distinguished when determining which one is the true relation held between two entities, thus neighboring relations are supposed to be ranked closely by embedding methods on the task of relation inference. After going through all the samples, we obtain a directed relation network. In order to measure the strength of connections between two relation nodes, we weight each directed edge by the ratio of outgoing edges from the source relation node to the target relation node over the total number of outgoing edges from the source relation node.

**Fig. 11:**
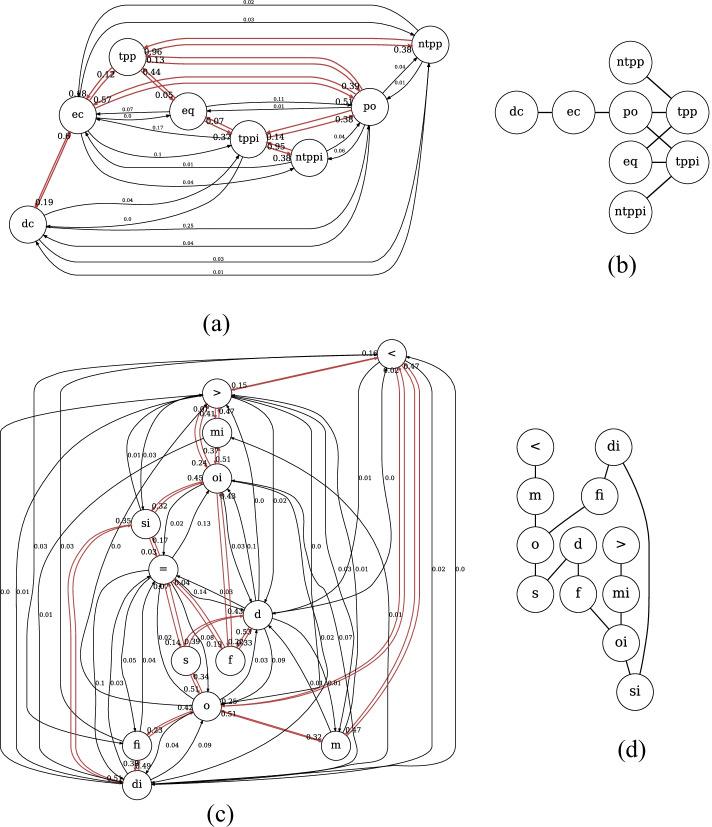
Conceptual neighborhood structures yielded by HyperQuaternionE

**Fig. 12:**
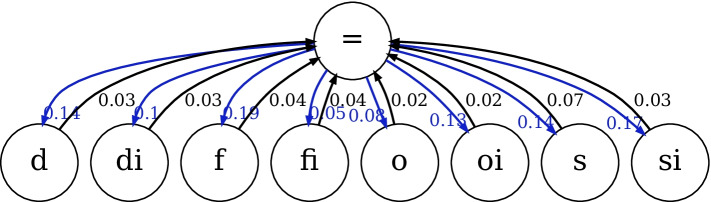
Original relation network around the relation $=$. Edges in blue are its outgoing edges while edges in black incoming

Figure 11 reveals original relation networks as well as conceptual neighbor structures yielded by HyperQuaternionE. Figure 11a and c are original relation networks, where nodes are spatial/temporal relations and the label on a directed edge is the strength of connections. Edges between two nodes are highlighted in red when the sum of weights in both directions is over threshold of 0.40^6^, which turns out to be neighborhood structures of relations shown in Fig. 11b and d after removing labels and arrows. In general, Fig. 11b and Fig. 10a are alike and Fig. 11d is similar to Fig. 10b. It indicates that our embedding method is capable of implicitly learning conceptual neighborhood structure of spatial/temporal relations. However, due to a lack of *equal* relations in both region187 and interval205^7^, it fails to completely reproduce the structure around *eq*/$=$. In addition, we find that for temporal relations another reason of failure for *equal* relation is that it has multiple conceptual neighbors and the proportion of outgoing edges to each target relation is marginal. Thereby, a relatively large threshold would easily filter out edges linked to the relation $=$ ( see Fig. 12). It reveals that our method successfully rules out four relations (i.e., <, *m*, *mi*, and >) that are impossible to be conceptual neighbors of the relation $=$ and learns that all the other eight relations can be transformed from it by differing proportions ($0.05-0.19$). This echos the neighborhood structures around relation $=$ in Fig. 10b.

**Fig. 13:**
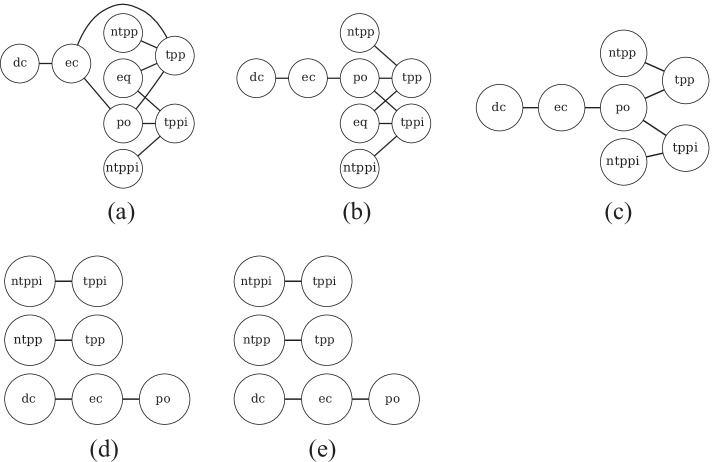
Network structures with varying thresholds (spatial relations)

**Fig. 14:**
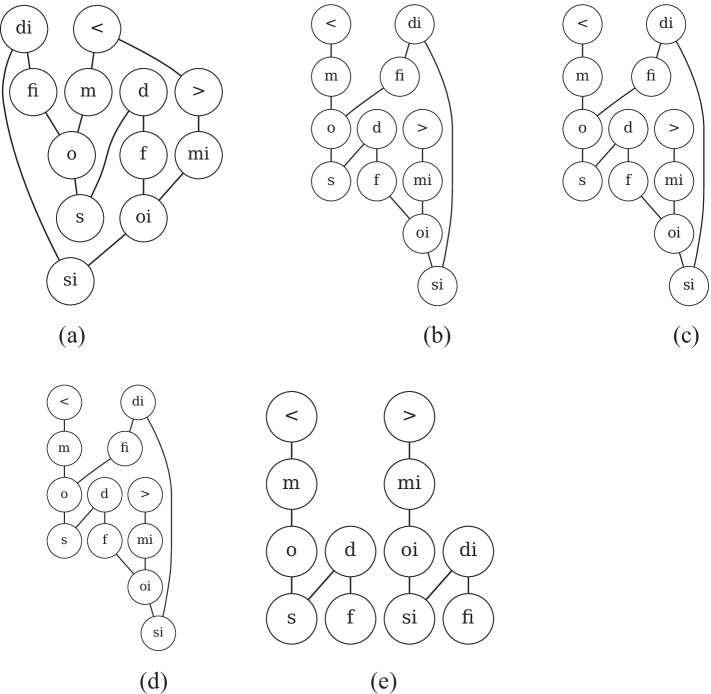
Network structures with varying thresholds (temporal relations)

Moreover, we set varying thresholds to investigate the closeness of neighboring relations. Figure 13 reveals that *nttpi-tppi* and *ntpp-tpp* are densely connected over changes, which is in line with the discovery of [9] that topological distances between them are the least. Furthermore, our method identifies another closely-connected chain: *dc-ec-po*, which intuitively makes sense as *ec* is the critical condition of continuous transformation between *dc* and *po*. Figure 14b, c and d confirm the stability of the found network structure between temporal relations. Meanwhile, it is interesting to see even when the threshold is set as large as 0.7 (meaning only edges with the strongest connections remains), two chain structures are recognized, where each relation and its inverse are separated in different chains.

**Fig. 15:**
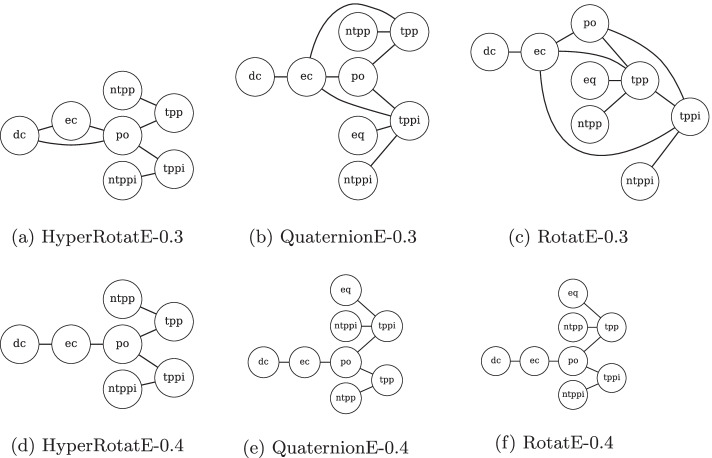
Network structures by different models (spatial relations)

**Fig. 16:**
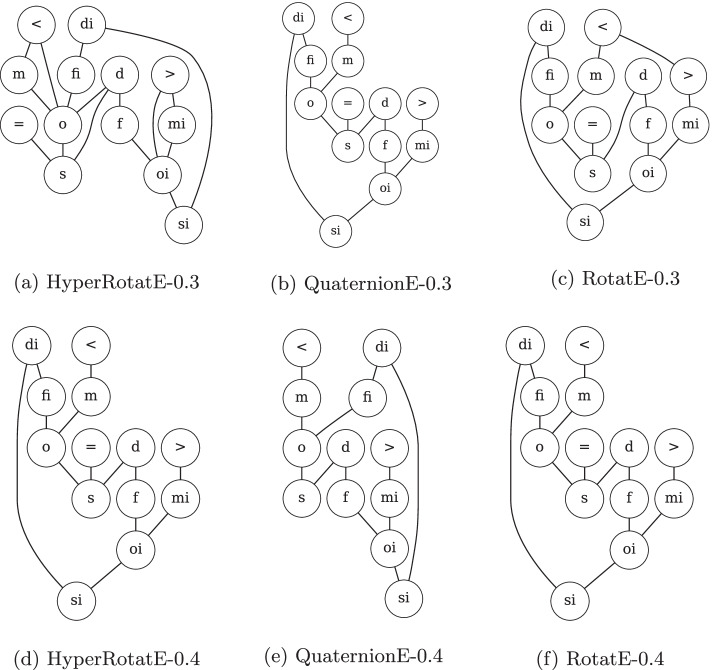
Network structures by different models (temporal relations)

Last but not least, we compare network structures of relations yielded by different embedding models (see Figs. 15 and  16 in Appendix). In general, results show that all embedding models are capable of implicitly learning neighborhood structures of relations with nuanced differences.

## Discussion and future work

Qualitative spatial and temporal reasoning [5, 19] have played a crucial role for a wide range of tasks such as topological integrity constraints in GIS, spatial queries, navigation and orientation in robotics, representing spatial human cognition, and so forth. Traditionally, composition tables of RCC-8 relations and temporal relations have been widely adopted in spatial reasoners to accomplish inference tasks. However, such symbolic reasoning with explicitly-injected knowledge has many restrictions that arise from the inability to efficiently deal with noise, missing data, high-order neighborhood information, or large datasets in general. This makes existing techniques unsuitable for many interesting applications, such as knowledge base completion and knowledge graph-based recommendation. Recently, success stories in Machine Learning (ML), in particular embedding techniques, shed light on spatial and temporal reasoning, thanks to their subsymbolic and learnable representations of knowledge. In this paper, we designed novel embedding-based methods for spatial and temporal reasoning and examined how these methods perform when compared against traditional methods. We were especially interested in examining whether embedding-based methods learn domain knowledge implicitly from data.

In order to answer these questions, we developed an embedding model, named as HyperQuaternionE. Our method is able to encode symmetric/anti-symmetric properties of relations and inverse relations, and can automatically find and capture composition patterns of relations from data, which is key to automatic spatial and temporal reasoning. Moreover, our method provides a hyperbolic embedding space to embed tree-like structures over entities induced by transitive relations such as *after* and *non-tangentially proper part*. We evaluated our work using two synthetic datasets (region187 and interval205), and compared different methods against relation inference and entity inference tasks. **The experimental results revealed that our embedding method achieves superior performance on both datasets in terms of both tasks and outperformed both other baseline embedding methods and the constraint network method relying on composition tables.**

We hypothesize that such strong results are partially because embedding methods are capable of capturing constraints from both local and global high-order information through training. Representations of entities and relations are learnable and updated globally over iterations. Another advantage of embedding methods lies in that they yield ranked lists of relations with high precision rather than sets of relations without order produced by traditional methods. A ranked list is more preferable, since in theory exactly one topological relation between two geographical entities holds due to the relations’ jointly exhaustiveness and pairwise disjoint (JEPD) characteristic. Moreover, we argued that embedding methods have much broader applications than traditional reasoners, such as entity inference and checking the validity of relations between two entities.

In order to answer the second research question, we analysed relation inference results and found that **embedding methods implicitly learned conceptual neighborhood structures of spatial relations and temporal relations, and some neighborhood structures are much more closely connected (such as** ***dc-ec-po*** and ***nttpi-tppi***) **than others.** This is a valuable discovery in two aspects. First, from the viewpoint of model interpretation, it helps explain why embedding methods succeed in spatial and temporal reasoning. Early on,  [19, 20] pointed out that the representation and/or reasoning processes will be considerably simplified by incorporating conceptually neighboring relations into reasoning. Second, from the viewpoint of model design, this suggests that understanding and analyzing what machine learning methods are able to learn from existing data is of great importance to theory-informed model design. For instance, with “enough” data available, as shown in our paper, conceptual neighbors of relations can be learned automatically and implicitly by models from data, and, thus, incorporating such theories/spatial thinking explicitly would not supply extra useful information.

Following the discussion above, this work raises several questions that deserve further investigation. First, in this paper we focused on the *qualitative* reasoning capability of embedding methods, and, thus, intuitively we assume the developed methods would not be affected by the original geometries of geographical entities. However, given that geographical entities with complex geometries (e.g., arbitrary polygons, polygons with holes, etc.) may bring about complex topological relations, it is worth examining the adaptability of embedding methods to such cases. Second, it is worth further exploring what other spatial theories or knowledge in spatial and temporal reasoning can be/have been learned implicitly in addition to conceptual neighborhood structures. This direction, broadly speaking, falls into the the bigger trend of *explainable AI* and ML in geography which is key for accountable data-driven decision making.

## Data Availability

Experimental data and the methods developed will be openly shared for reproducibility and replicability https://github.com/ling-cai/hyperQuaternion-spatiotemporal-reasoning.

## Appendix A: Some proofs

### A.1 Unitary quaternion

25 $$\begin{aligned} q_u=cos(\alpha ) + sin(\alpha )cos(\theta _1)cos(\theta _2)i + sin(\alpha )cos(\theta _1)sin(\theta _2)j+ sin(\alpha )sin(\theta _1)k \end{aligned}$$

$$\begin{aligned} \Vert q_u \Vert= & {} \sqrt{cos(\alpha )^2+(sin(\alpha )cos(\theta _1)cos(\theta _2))^2+(sin(\alpha )cos(\theta _1)sin(\theta _2))^2 + (sin(\alpha )sin(\theta _1))^2} \\= & {} \sqrt{cos(\alpha )^2+(sin(\alpha )cos(\theta _1))^2+(sin(\alpha )sin(\theta _1))^2} \\= & {} \sqrt{cos(\alpha )^2+sin(\alpha )^2} =1. \end{aligned}$$

## Appendix B: Number of learnable parameters

Table 10.

**Table 10:** Number of parameters in each model. |*E*| and |*R*| are number of entities and relations, respectively

Model	#parameters
HyperQuaternionE	$d*|E| + (d+1)* |R|$
HyperRotatE	$(d+1)*|E|+(3d+1)*|R|$
QuaternionE	$d*|E|+d*|R|$
RotatE	$2d*|E|+d*|R|$

## Appendix C: Network structures by different embedding models

Here we compare network structures yielded by different embedding models. Note that since there is no practical guideline on how to determine thresholds to extract closely-connected substructures, we choose threshold=0.3 and threshold=0.4 empirically. In general, according to Figs. 15 and 16, we can conclude that all embedding models are capable of implicitly learning neighborhood structures of relations with nuanced differences. By comparing Fig. 15d, e, f with Fig. 13b, we can find that our model yields a better structure as part of the substructure around *eq* is discovered successfully while others fail to do so. For network structures of temporal relations (Fig. 16e, e and 14b), they all exhibit much similarity except for small differences around $=$, which is partly attributed to a lack of *equal* relations in datasets. However, as discussed in Section 5.4.4, our model in fact discovers the inner structure around $=$, which is filtered out by thresholds yet. Therefore, our model is superior to other embedding models in discovering relationships between relations.

## Footnotes

1 For instance, if entity A is non-tangential proper part of entity B and entity B is externally connected to entity C, then entity A must be disconnected to entity C.

2 We omited TransE here, since its performance is relatively weak.

3 https://github.com/alreich/qualreas

4 For example, for a test query (geometry 1, dc, geometry 2), it is expected that a model should output *geometry 2* as the correct answer to a query (geometry 1, dc, ?t). However, there may exist other geometries in the KG that can satisfy the query. In such cases, the model should not be penalized if other valid geometries are ranked ahead of geometry 2.

5 Usually a training process needs more time when the hidden dimension is high.

6 This threshold is chose empirically. We also report results when thresholds vary in Figs. 13 and 14.

7 There are only 192 triples with *eq* relation (187 of which is self-equal (e.g., $<h, eq, h>$)) and 210 triples with $=$ relation (205 of which is self-equal).
